# Supplementary material for: Direct identification of interfacial degradation in blue OLEDs using nanoscale chemical depth profiling
Source: Nat Commun. 2023 Dec 6;14:8066. doi: 10.1038/s41467-023-43840-9 (PMC10698160; doi:10.1038/s41467-023-43840-9)
Supplement: Supplementary file 1 — Supplementary Information [file 41467_2023_43840_MOESM1_ESM.pdf]

Supplementary Information

# Direct identification of interfacial degradation in blue OLEDs using nanoscale chemical depth profiling

Gustavo F. Trindade[1§], Soohwan Sul[2§], Joonghyuk Kim[2§], Rasmus Havelund[1§], Anya Eyres[1], Sungjun Park[2], Youngsik Shin[2], Hye Jin Bae[2], Young Mo Sung[2], Lidija Matjacic[1], Yongsik Jung[2], Jungyeon Won[2], Woo Sung Jeon[2], Hyeonho Choi[2], Hyo Sug Lee[2], Jae-Cheol Lee[2+], Jung-Hwa Kim[2*] and Ian S. Gilmore[1*]

§ These authors contributed equally

[1] National Physical Laboratory, Hampton Road, TW11 0LW, United Kingdom

[2] Samsung Advanced Institute of Technology, Samsung Electronics Co., Ltd., 130 Samsung-ro, Suwon, 16678, Republic of Korea

[+] Present address: Korea Research Institute of Material Property Analysis (KRIMPA), 712, Nongseo-dong 455, Yongin, 17111, Republic of Korea

*Corresponding authors

*E-mail: jh1179.kim@samsung.com

*E-mail: ian.gilmore@npl.co.uk,

## Contents

Supplementary Note 1: Further characterisation of device A ........ 2

Supplementary Note 2: Optimisation of gas cluster ion beam sputtering conditions to study OLED degradation products ........ 7

Supplementary Note 3: Estimation of blue dopant degradation using photoluminescence measurements ... 9

Supplementary Note 4: Full NMF results related to Figure 2 of main text ........ 12

References ........ 19

# Supplementary Note 1: Further characterisation of device A

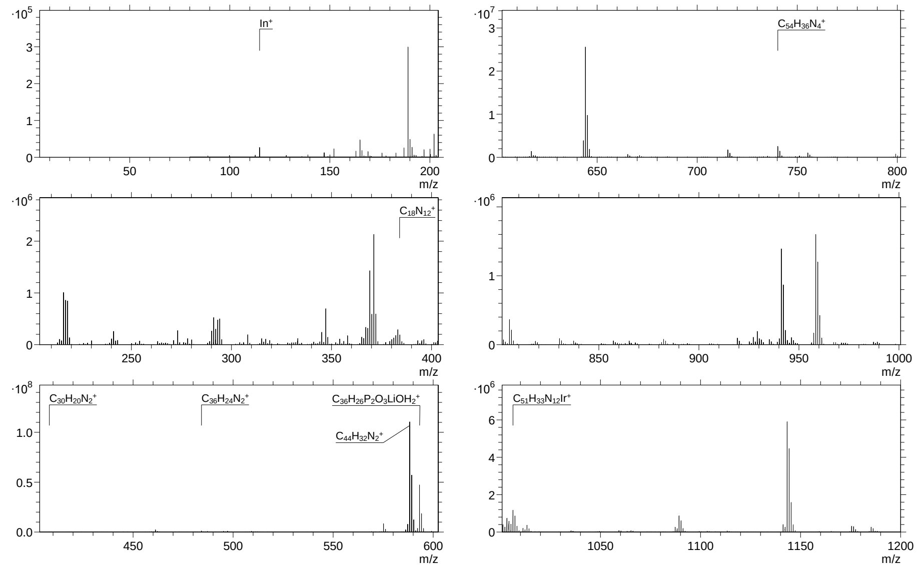


**Supplementary Figure 1: Full mass spectrum from pristine device A.** The mass spectrum represents an overall depth profile acquired using 5 keV $Ar_{2000}^+$ cluster primary ion beam.

**Supplementary Table 1.** Characteristic molecular ions in the depth profile of the devices.

| ***m/z*** | **Assignment** | **Description** | **Mass Deviation (ppm)** |
|---|---|---|---|
| 114.9032 | $In^+$ | ITO | -1.3 |
| 384.0364 | $C_{18}N_{12}^+$ | HAT-CN | 0.1 |
| 408.1618 | $C_{30}H_{20}N_2^+$ | mCP | -0.6 |
| 484.1934 | $C_{36}H_{24}N_2^+$ | mCBP (Host 1) | 0.0 |
| 509.1885 | $C_{37}H^{23}N_3^+$ | mcBP-CN (Host 2) | -0.4 |
| 537.1852 | $C_{36}H_{29}SiPOH^+$ | TSPO1 | -1.5 |
| 588.2549 | $C_{44}H_{32}N_2^+$ | NPB | -1.9 |
| 593.1610 | $C_{36}H_{26}P_2O_3LiOH_2^+$ | DBFPO:Liq | -1.3 |
| 740.2932 | $C_{54}H_{36}N_4^+$ | TCTA | -0.3 |
| 1006.2571 | $C_{51}H_{33}N_{12}Ir^+$ | Dopant 1 | -0.9 |
| 1096.4446 | $C_{78}H_{54}B_2N_6^+$ | v-DABNA (Dopant 2) | -0.7 |
| 561.2035 | $C_{36}H_{29}SiPOHLiOH^+$ | TSPO1:Liq | -3.3 |
| 534.1867 | $C_{38}H_{22}N_4^+$ | mcBP-$(CN)_2$ (Host 3) | -1.5 |

**Supplementary Table 2.** Peak list from MS/MS mass spectrum from a pristine device **A** (precursor m/z 1006.2569)

| | | | | | | | |
|---|---|---|---|---|---|---|---|
| **m/z** | 112.7207 | 112.7265 | 273.1134 | 303.1560 | 303.1601 | 345.1135 | 347.1290 |
| **m/z** | 348.1325 | 540.0919 | 732.1354 | 734.1511 | 743.1411 | 744.1444 | 751.1538 |
| **m/z** | 764.1254 | 766.1409 | 782.1357 | 808.1665 | 838.1758 | 856.1863 | 927.2026 |
| **m/z** | 929.2180 | 1002.2346 | 1003.2334 | 1004.2410 | 1005.2492 | 1006.2569 | |

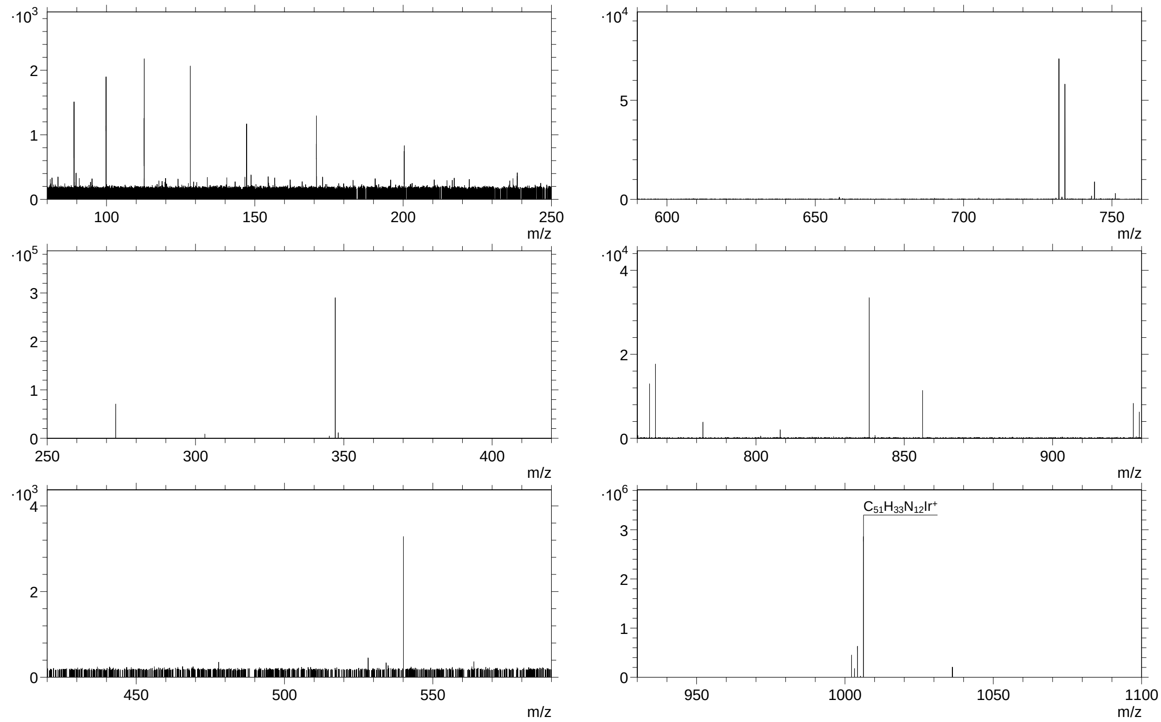


**Supplementary Figure 2: MS/MS mass spectrum from *m/z* 1006.2569**. In order to achieve a collision induced dissociation of the host material, pre-sputtering was performed to reach the EML. The MS/MS analysis was then undertaken using $N_2$ gas to fragment at 20 NCE (Nominal Collison Energy). Both the pre-sputtering and MS/MS were acquired using 5 keV $Ar_{2000}^+$ cluster primary ion beam.

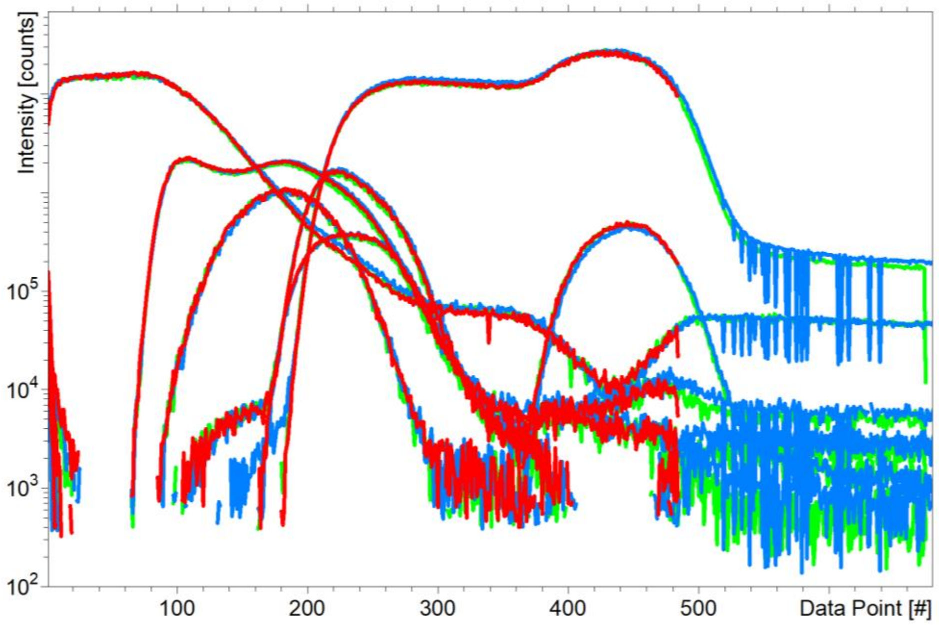


**Supplementary Figure 3**: **Technical repeatability with three repeat depth profiles recorded from different areas of the same device of type A**. The profiles are essentially indistinguishable from each other demonstrating good measurement repeatability.

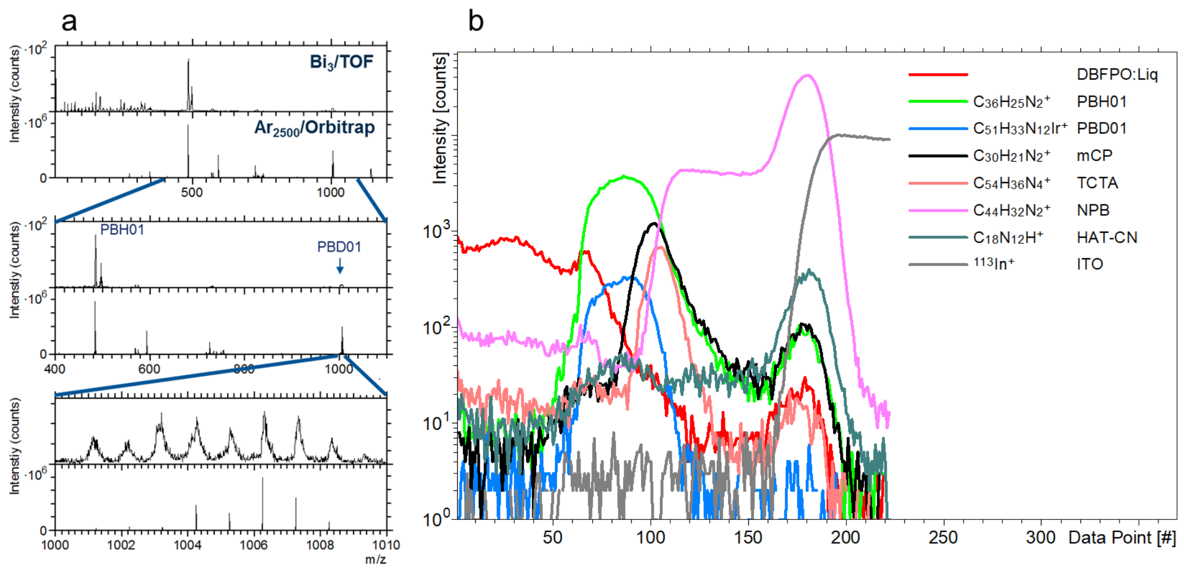


**Supplementary Figure 4: Comparison between Orbitrap and tof analysers for depth profiling a blue OLED**. **a** Comparison of spectra obtained from the emissive layer in a device using the $Bi_3^+$/ToF and the $Ar_{2500}^+$/Orbitrap. **b** Depth profile obtained from a degraded device using $Bi_3^+$ primary ions and the ToF mass analyser.

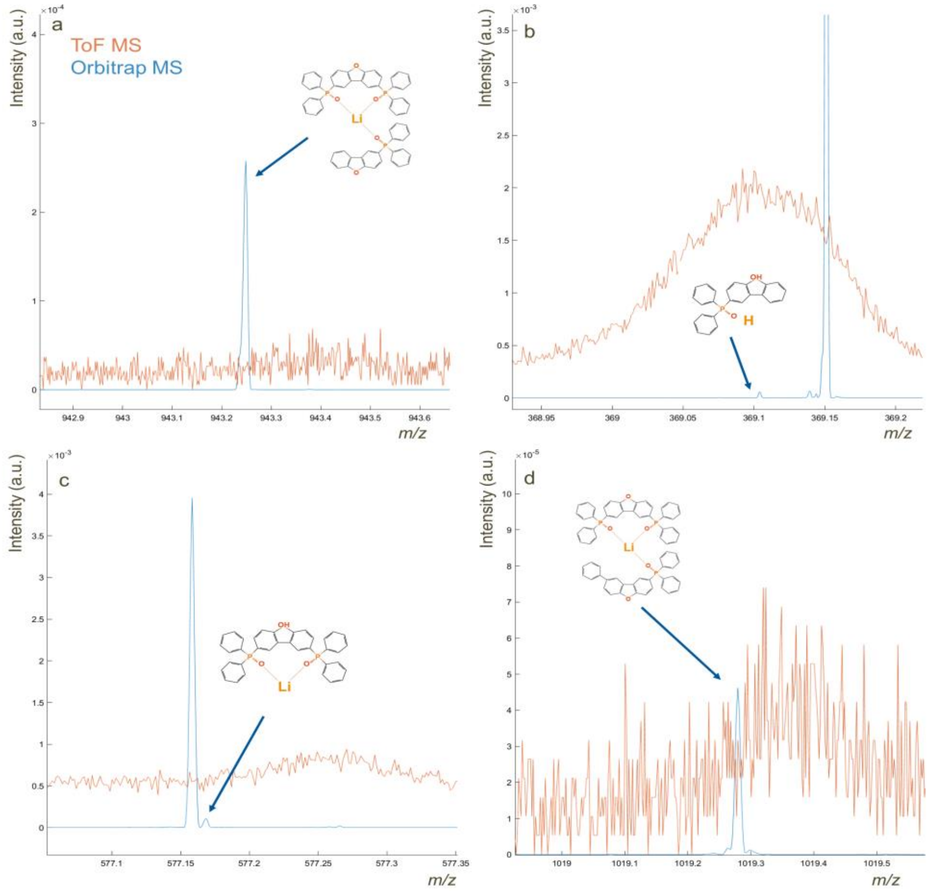


**Supplementary Figure 5: Comparison of spectra of degradation products by Orbitrap and ToF analysers. a-d** Comparison of spectra using $Bi_3^+$/ToF and $Ar_{2500}^+$/Orbitrap showing the peaks of 4 blue phosphorescent OLED degradation products.

**Supplementary Table 3 .** Electrical degradation of the blue OLED device and device characteristics (Type A). Performance characteristics at 1,000 cd $m^{-2}$ is given as electroluminescence (EL) peak position, driving voltage ($V_d$), current density (J) at 5 V, current efficiency (cd $A^{-1}$), maximum external quantum efficiency (Max EQE) and EQE.

| Device label | Luminance relative to initial | EL peak (nm) | Voltage ($V_d$) | J at 5 V (mA $cm^{-2}$) | Current efficiency (cd $A^{-1}$) | Max EQE (%) | EQE (%) |
|---|---|---|---|---|---|---|---|
| T100 | Pristine (100% EL) | 469 | 5.9 | 0.6 | 26.1 | 19.9 | 18.6 |
| T90 | 90% EL | 469 | 6.0 | 0.6 | 24.1 | 17.8 | 17.1 |
| T70 | 70% EL | 469 | 6.2 | 0.5 | 18.9 | 13.7 | 13.4 |
| T50 | 50% EL | 470 | 6.5 | 0.4 | 13.8 | 9.9 | 9.8 |

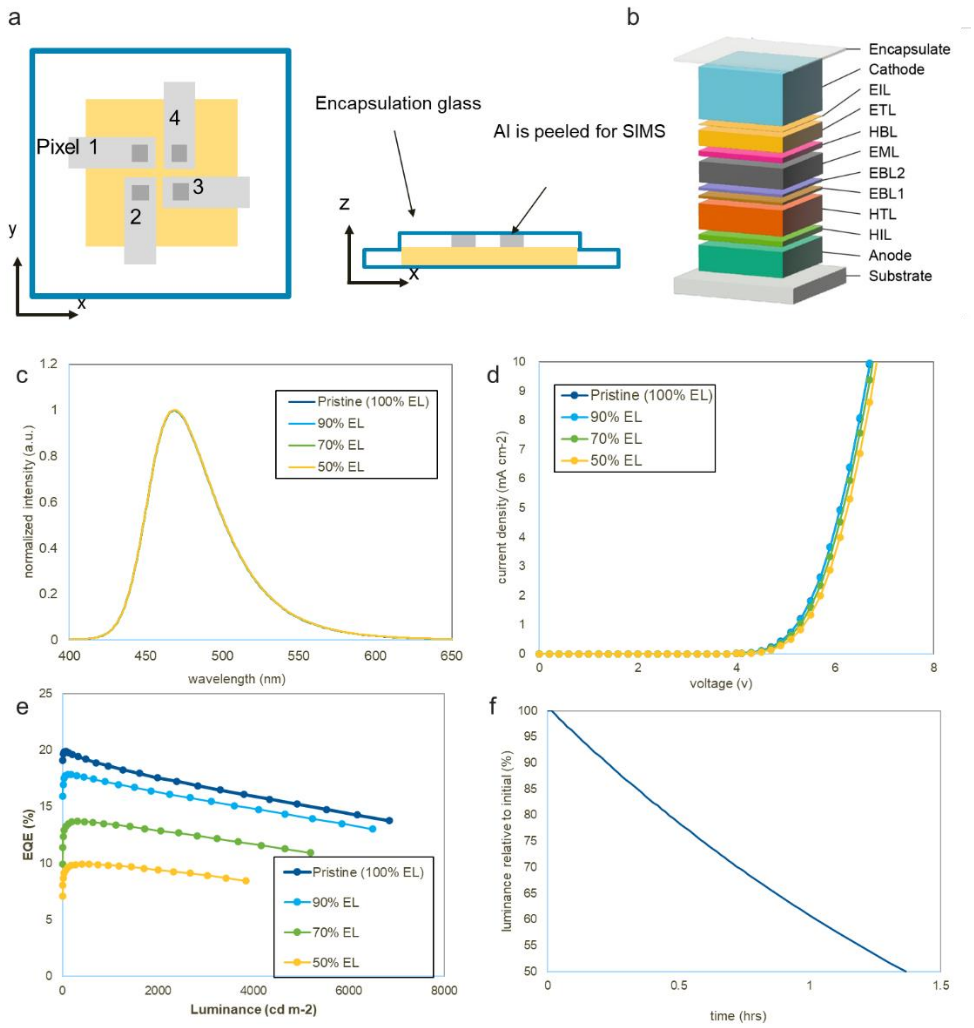


**Supplementary Figure 6. Electronic characterisation of a blue OLED device. a** Device schematic showing 4 pixels that are each degraded to a different level. **b** Device layered structure. EL spectra **c** current density vs. voltage plot **d** External quantum efficiency as a function of luminance **e**, and **f** electroluminescence decay as a function of time for Type A device.

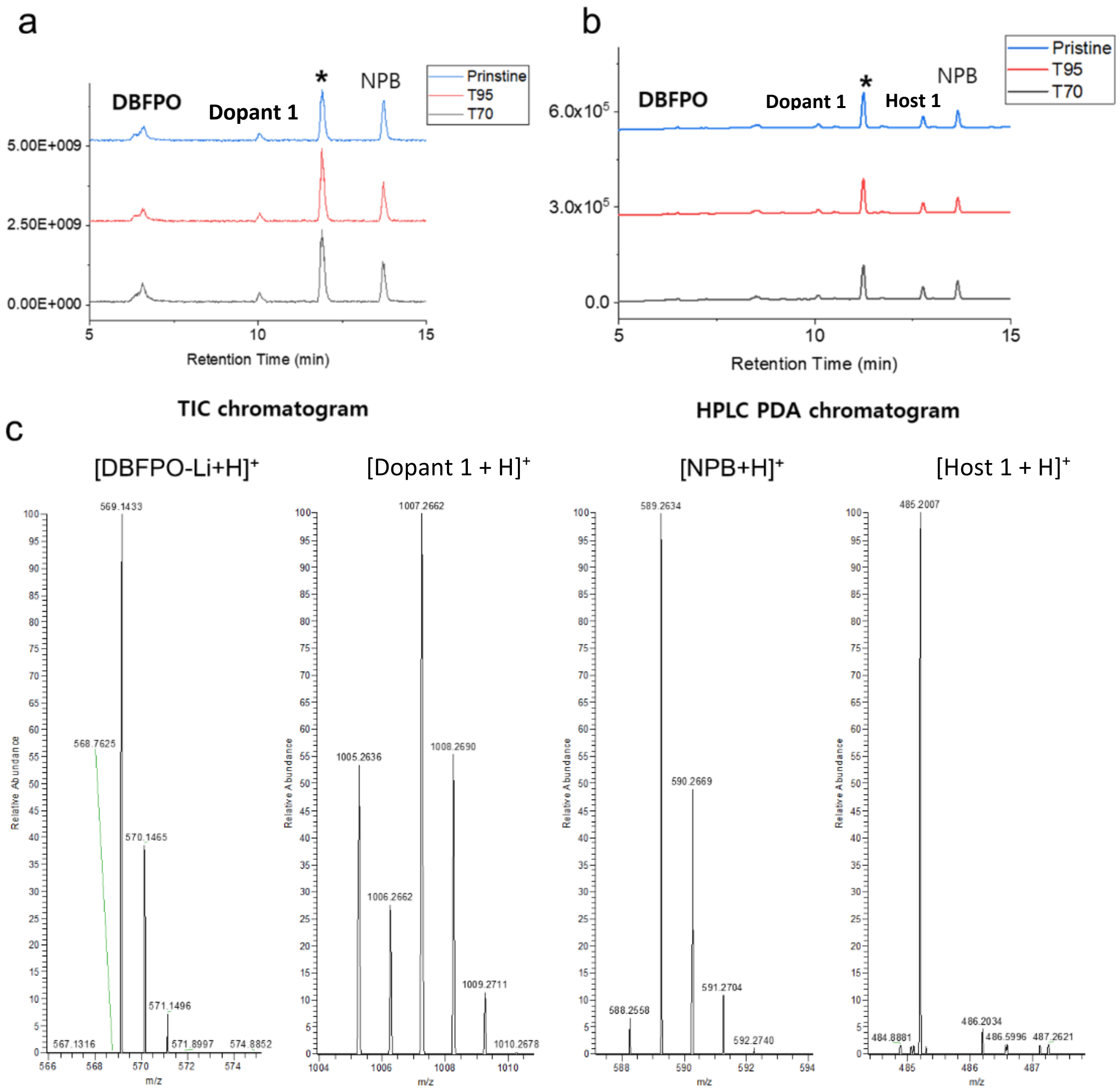


**Supplementary Figure 7: LC-MS analysis. a** LC-MS chromatogram (total ion current). **b** HPLC PDA chromatogram * impurity. **c** Corresponding mass spectra at specific retention times

## Supplementary Note 2: Optimisation of gas cluster ion beam sputtering conditions to study OLED degradation products

To optimise the detection sensitivity of degradation products at the ETL/EML interface we minimised degradation induced by the gas cluster sputtering beam. To do this, we measured $T_{100}$ and $T_{70}$ devices of type **B, E and F (**as described in **Table 3** of the main text) varying the mean energy per atom in the primary gas cluster ion beam. The measured beam cluster size distribution for each ion beam used are shown in **Supplementary Figure 8**.

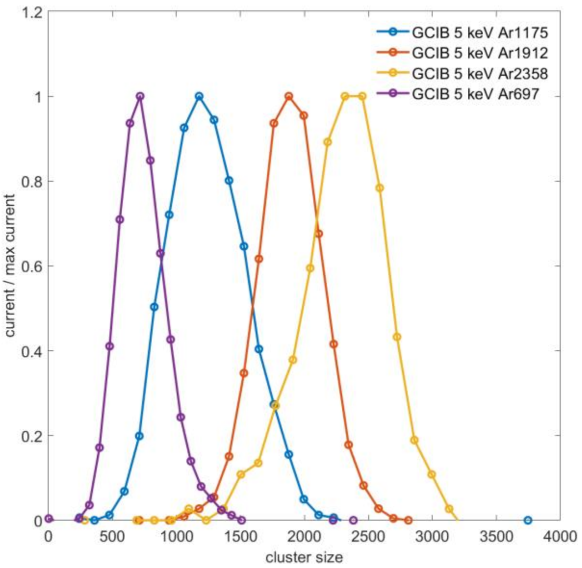


**Supplementary Figure 8**: **Measured beam cluster size distribution for each ion beam used to study beam-induced degradation of the OLED devices.**

**Supplementary Figure 9a** shows the normalised intensities of the 5 degradation products described in **Tables 2 and 3** of the main text in function of the eV/atom of the primary ion beam used, measured across 6 different devices. The intensities are normalised by the DBFPO:Liq signal. **Supplementary Figure 9b** shows the depth profiles of the degradation product represented by the ion $C_{24}H_{18}PO_2^+$ for a device of type **C** (Host 2, Dopant 1) with all 4 different ion beams. The results show that ion beams with higher mean energy per atom (e.g. 5 keV $Ar_{697}^+$) will induce the formation of the same DBFPO degradation products at the ETL/EML interface for any device, pristine or driven to a certain lifetime stage (e.g. $T_{70}$) and would hinder the study of degradation resulting from the induced degradation to $T_{70}$, $T_{95}$ and $PL_{55}$ levels, which have very low signal intensity. **Supplementary Figure 10** shows the results separately for a $T_{100}$ and a $T_{70}$ device of type **C** (Host 2, Dopant 1). At 2 eV/atom, there is little difference between $T_{70}$ and $T_{100}$, which would represent a more stable device in contrast to device type **A** studied and described in the main text. At higher eV/atom, the signal of the degradation product $C_{24}H_{18}PO_2^+$ becomes increasingly higher for $T_{70}$ in relation to $T_{100}$. This could be used as an additional metric to establish usage level or stability of a device, however, the results are not consistent for all ions or devices and more investigation must be done. Nevertheless, this study of ion beam-induced degradation shows that the most appropriate condition to study organic molecules in a sensitive device such as a blue OLED is a primary ion beam of below 2 eV / atom.

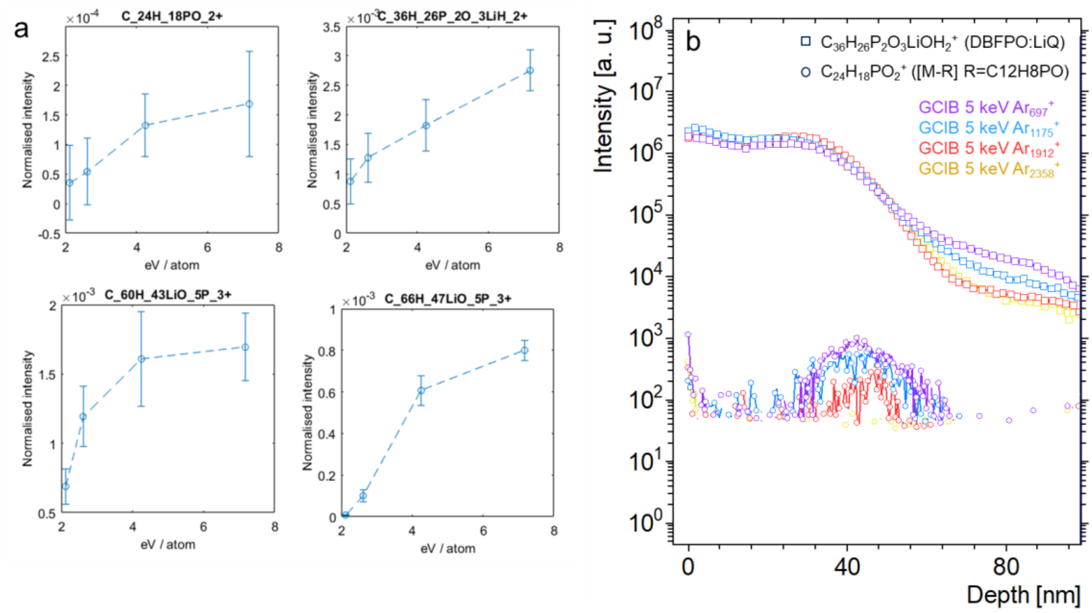


**Supplementary Figure 9**: **Intensity of degradation products as a function of beam cluster size. a** Normalised intensities of the 5 degradation products described in **Tables 2 and 3** of the main text in function of the eV/atom of the primary ion beam used, measured across 6 different devices. The intensities are normalised by the DBFPO:Liq signal. **b** Depth profiles of the degradation product **b** Depth profiles of selected ions $C_{36}H_{26}P_2O_3LiOH_2^+$ (squares) and $C_{24}H_{18}PO_2^+$ (circles) for the different ion beam energies for a device of type **C** (Host 2, Dopant 1). Error bars correspond to the standard deviation of 3 measurements.

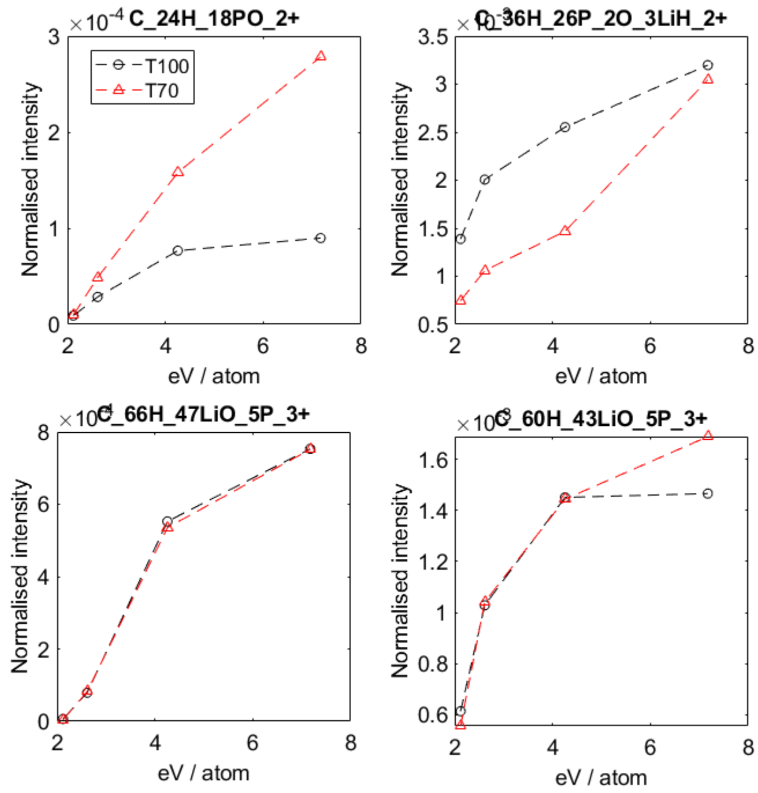

**Supplementary Figure 10**: **Normalised intensities of the 5 degradation products described in Tables 2 and 3 of the main text in function of the eV/atom of the primary ion beam used**, showed separately for a T100 and a T70 device of type **C** (Host 2, Dopant 1).

## Supplementary Note 3: Estimation of blue dopant degradation using photoluminescence measurements

A device of type **A** in **Table 3** of the main text ($PL_{55}$) was degraded under irradiation of 400-nm laser until a 55% photoluminescence level (PL) was reached. We performed photoluminescence (PL) measurements for a comparative quantitative analysis on blue emitting dopant degradation of type A (**Supplementary Figure 11**). Whereas electroluminescence (EL) decreased to 95% and 70% in $T_{95}$ and $T_{70}$, respectively, the PL intensity was 98.2% and 92.9 % from the corresponding devices. This indicates that the device degradation in the aged devices $T_{95}$ and $T_{70}$ does not mainly originate from blue Ir dopants, which contributes only 7% to the total luminescence loss of the device $T_{70}$.

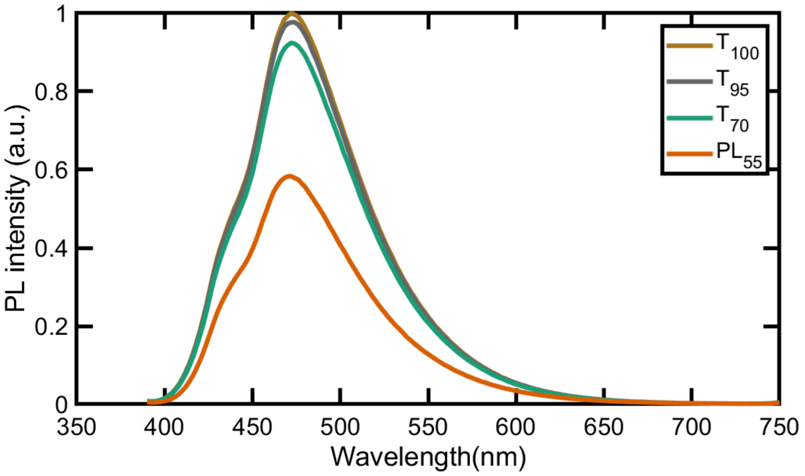


**Supplementary Figure 11**: **Photoluminescence spectra of Type A device at different electro-degradation levels.**

Considering the wavelength of the laser and energy levels of layer materials[1], HTL (NPB, 3.1 eV), blue Ir dopant (2.9 eV), and ETL (DBFPO:Liq, 3.4 eV) may absorb the intense light, which leads to material degradation through high energy exciton generation via exciton-exciton annihilation or photo-thermal effect.[2] We also performed unsupervised multivariate analysis[3] of a combined dataset with depth profiles for each device to identify thermal degradation products in the NPB layer of the $PL_{55}$ device, which are likely induced by the laser irradiation[4] (**Supplementary Figures 12-13**).

Unsupervised multivariate analysis was carried out using secondary ion masses as the variables and depth levels as observations. For each dataset, Surface Lab 7.3 (IONTOF GmbH) was used to perform an automated peak search on the total spectra restricted only to peaks with intensity higher than $10^5$ and lower than $10^9$ with masses between *m*/*z* 200 and *m*/*z* 1200. Peak areas were then exported for each observation. Principal component analysis (PCA) was performed using the simsMVA software[3] on a joint dataset containing all depth levels for the profiles of all devices as observations. Prior to PCA, data was mean-centred and Poisson scaled to account for non-uniform noise across mass spectrum. PCs 1-3 have very similar scores for all devices and their loadings can be attributed to device architecture identified in the main text. PC 4 is the only component that shows PL55 has an enhancement of thermal degradation product NPB-N

$C_{34}H_{27}N_2^+$ in the NPB layer of the PL55 device, which is likely induced by the laser irradiation[4] (**Supplementary Figure 12a-c).** The full PCA results PCs1-4 are in **Supplementary Figure 13**.

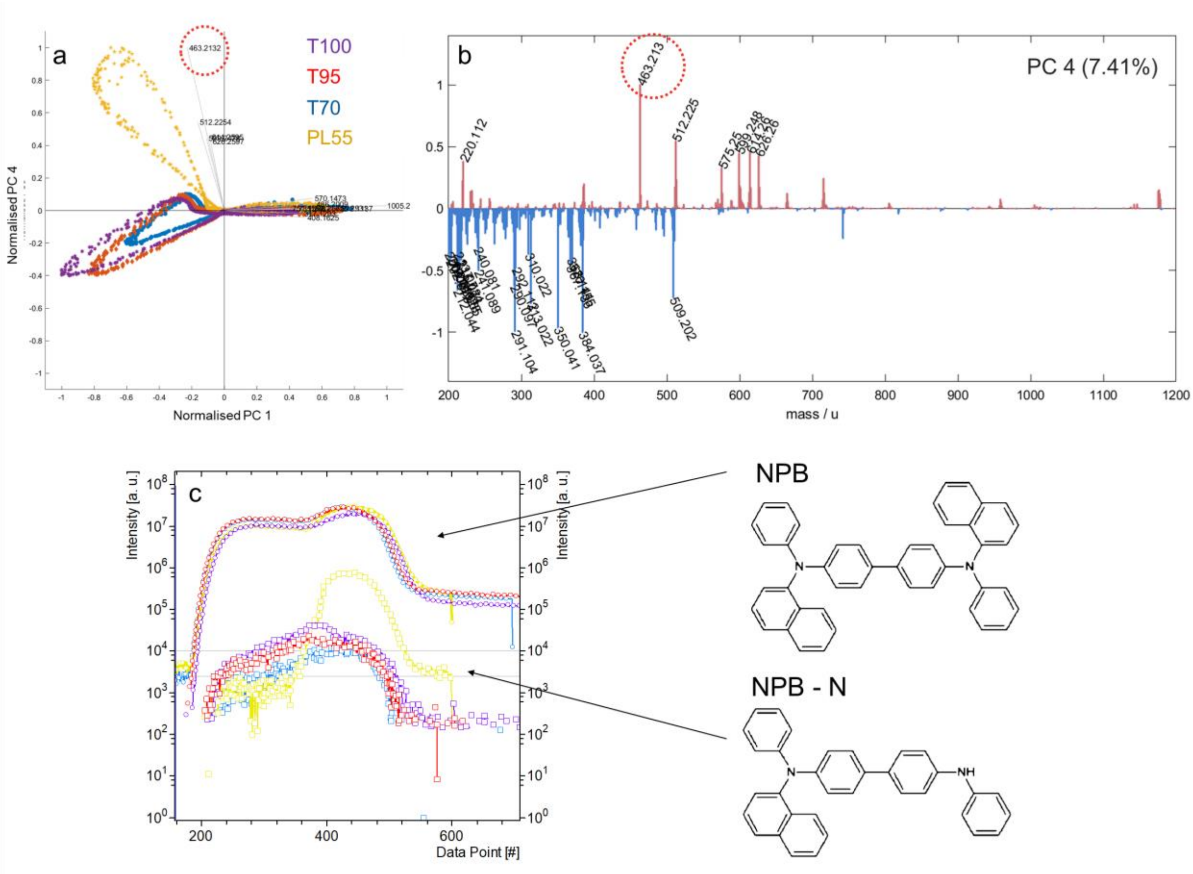


**Supplementary Figure 12**: **Principal component analysis of OrbiSIMS depth profiles of pristine and degraded blue OLEDs. a** Scatter biplot of principal components 1 and 5 analysis with points colour coded based on device depth profile. **b** Loadings of PC5. The red circle shows the NPB-N ion that is more intense for the PL55 device, indicating thermal degradation. **c** Overlaid depth profiles for each device showing the NPB molecular ion and the NPB-N ion enhanced for the PL55 device (yellow curves).

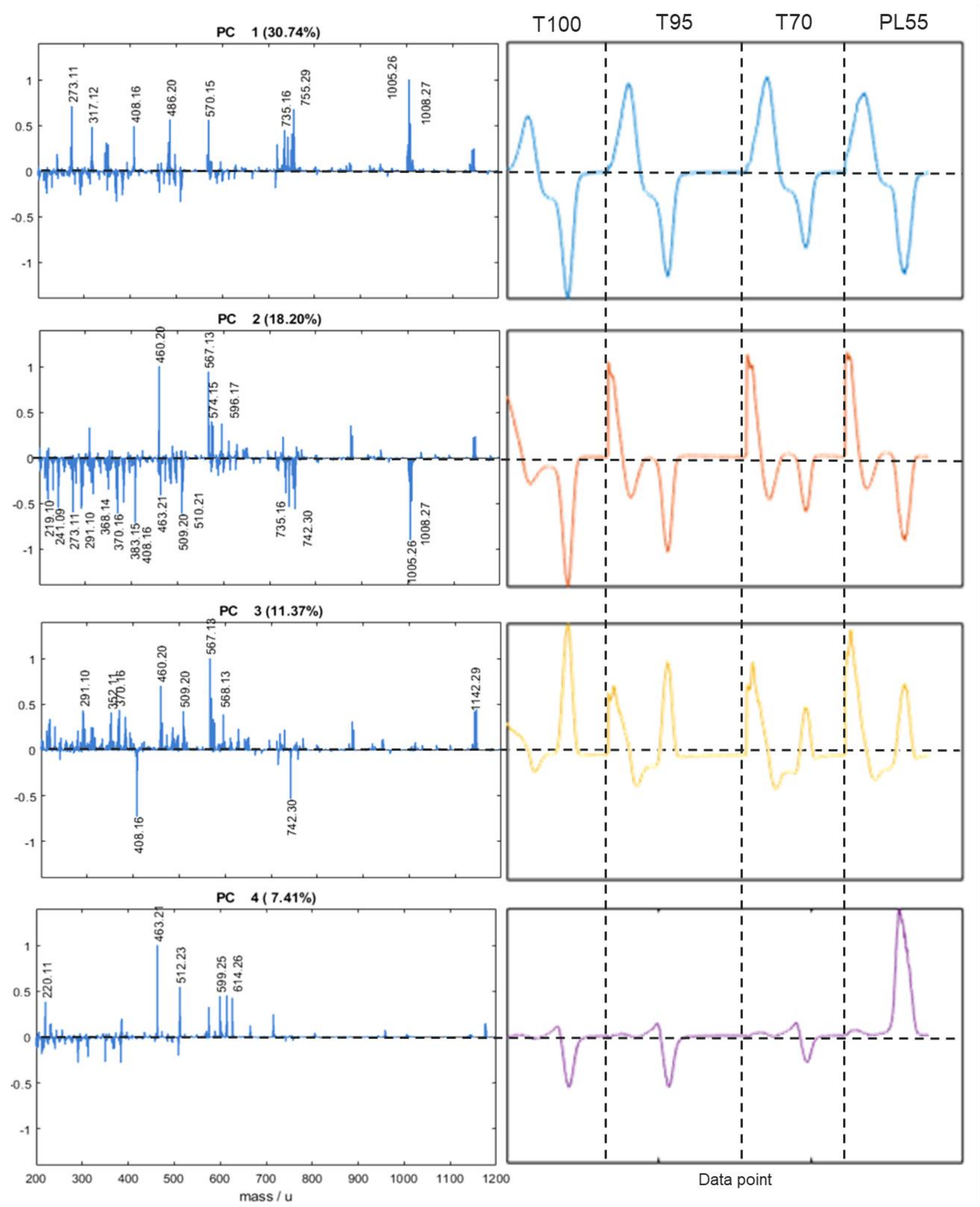


**Supplementary Figure 13**: **PCA results for joint OrbiSIMS depth profiles of blue OLEDs.** PCA loadings and scores for PC 1-4 of a joint dataset with 4 depth profiles.

# Supplementary Note 4: Full NMF results related to Figure 2 of main text

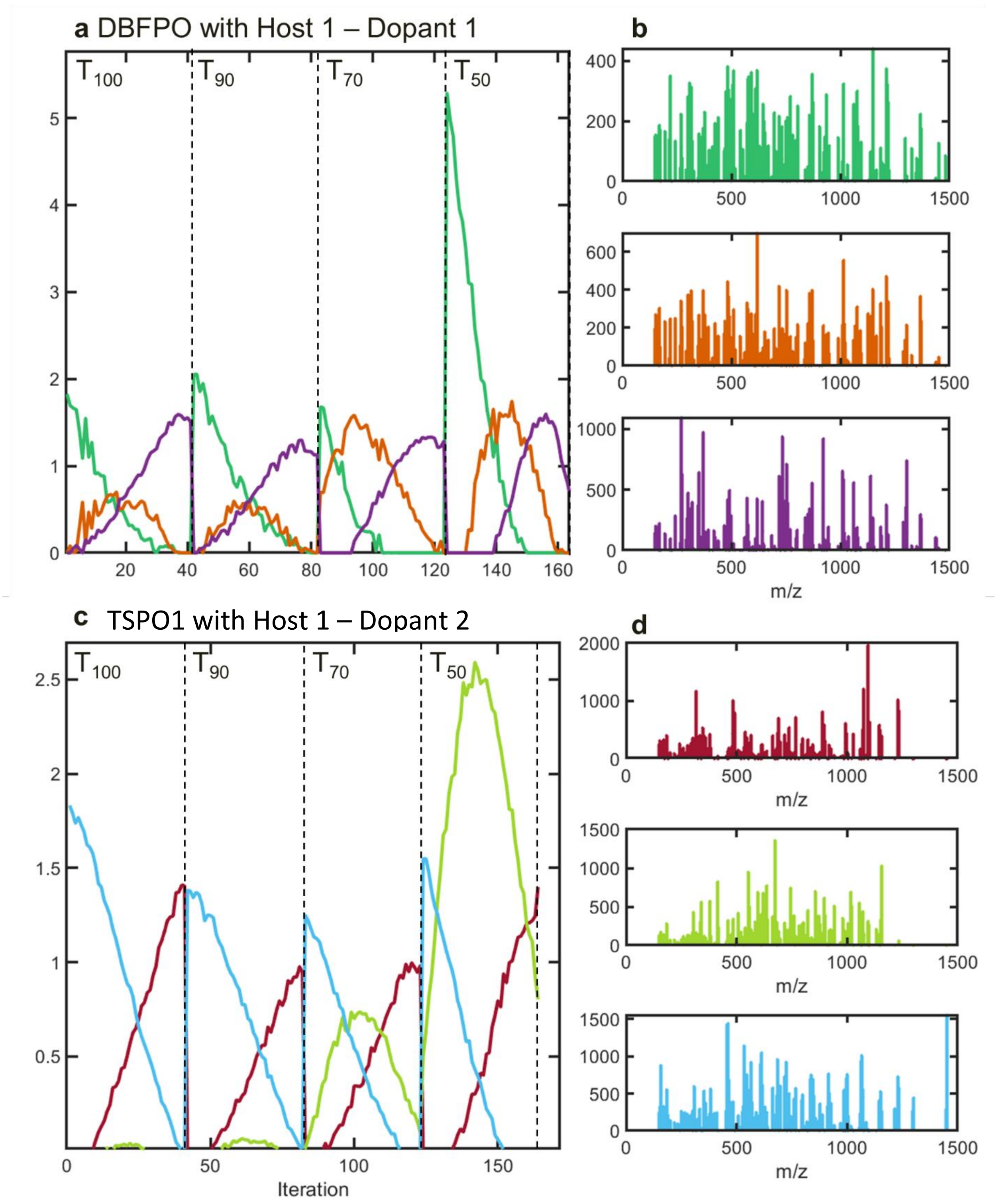


**Supplementary Figure 14**: **Non-negative matrix factorisation (NMF) of joint datasets of blue OLEDs at the HBL/EML interface.** NMF results for the joint datasets with $T_{100}$ to $T_{50}$ of DBFPO (**a-b**) and TSPO1-based (**c-d**) devices.

**Supplementary Table 4**: NMF factor loadings for the joint datasets T100 to T50 of DBFPO and TSPO1-based devices.

| **DBFPO with Host 1 - Dopant 1** | | | | **TSPO1 with Host 1 - Dopant 2** | | | |
|---|---|---|---|---|---|---|---|
| ***m/z*** | **Factor 1** | **Factor 2** | **Factor 3** | ***m/z*** | **Factor 1** | **Factor 2** | **Factor 3** |
| 150.0593 | 149.3 | 196.6 | 143.8 | 150.0593 | 254.4 | 129.8 | 248.3 |
| 152.0619 | 119.0 | 156.8 | 109.2 | 152.0636 | 315.8 | 135.7 | 319.4 |
| 152.0637 | 154.4 | 270.1 | 199.9 | 155.0853 | 104.7 | 98.1 | 121.9 |
| 157.0754 | 110.9 | 90.3 | 61.7 | 157.0314 | 259.6 | 171.9 | 243.2 |
| 167.0554 | 1.0 | 0.0 | 0.0 | 157.0753 | 119.9 | 164.5 | 877.5 |
| 168.0699 | 123.3 | 223.1 | 161.1 | 159.0796 | 25.1 | 91.7 | 483.0 |
| 170.0741 | 186.0 | 303.9 | 222.2 | 168.0699 | 253.4 | 105.1 | 238.2 |
| 173.0513 | 171.5 | 100.2 | 0.0 | 170.0741 | 334.9 | 140.4 | 326.8 |
| 175.1028 | 0.0 | 1.2 | 0.0 | 174.0849 | 0.0 | 19.5 | 161.6 |
| 194.0974 | 165.9 | 233.6 | 138.1 | 180.0387 | 70.4 | 13.4 | 110.9 |
| 217.0413 | 141.1 | 43.9 | 0.0 | 183.0356 | 407.2 | 280.7 | 552.5 |
| 220.0602 | 350.1 | 247.7 | 96.3 | 190.0660 | 0.0 | 36.9 | 210.9 |
| 225.0428 | 0.1 | 0.4 | 0.0 | 199.0572 | 112.7 | 69.4 | 141.1 |
| 227.0221 | 0.0 | 0.0 | 0.0 | 218.0710 | 96.2 | 72.1 | 113.8 |
| 241.0885 | 94.6 | 194.4 | 159.9 | 234.0661 | 55.0 | 41.0 | 95.3 |
| 242.0964 | 134.1 | 250.3 | 284.0 | 242.0962 | 265.4 | 23.7 | 0.0 |
| 267.0945 | 46.2 | 109.1 | 64.6 | 249.0838 | 103.9 | 72.9 | 264.7 |
| 268.0522 | 223.2 | 341.3 | 645.7 | 259.0670 | 55.3 | 71.4 | 150.7 |
| 271.0978 | 114.3 | 288.4 | 1092.7 | 259.0933 | 191.4 | 117.6 | 190.7 |
| 272.1010 | 1.2 | 0.0 | 150.5 | 266.0934 | 84.9 | 82.7 | 243.3 |
| 273.0463 | 78.5 | 19.5 | 0.0 | 268.0976 | 71.1 | 50.0 | 168.7 |
| 274.1168 | 67.1 | 205.8 | 626.0 | 275.0884 | 128.7 | 85.6 | 179.0 |
| 291.1041 | 37.7 | 76.5 | 88.3 | 277.0774 | 64.3 | 102.0 | 224.2 |
| 295.1052 | 2.8 | 61.9 | 261.3 | 278.1037 | 235.3 | 131.9 | 206.2 |
| 300.1055 | 120.7 | 124.6 | 88.5 | 279.1010 | 106.0 | 76.0 | 112.1 |
| 301.1136 | 281.5 | 374.2 | 472.5 | 285.1012 | 62.5 | 131.2 | 8.5 |
| 302.1215 | 0.0 | 120.7 | 345.1 | 295.1148 | 278.0 | 125.3 | 217.0 |
| 307.1217 | 326.3 | 200.7 | 167.7 | 300.1055 | 91.7 | 28.1 | 120.4 |
| 308.1363 | 63.2 | 38.9 | 24.4 | 300.1953 | 39.5 | 21.3 | 161.0 |
| 310.1406 | 49.4 | 20.0 | 15.5 | 301.1133 | 332.5 | 53.8 | 269.7 |
| 315.1040 | 313.9 | 395.7 | 319.6 | 302.1109 | 62.6 | 169.5 | 9.2 |
| 316.1120 | 219.1 | 336.4 | 348.8 | 302.1211 | 363.5 | 49.3 | 0.0 |
| 318.1234 | 135.3 | 237.1 | 303.8 | 304.1150 | 248.0 | 428.9 | 139.2 |
| 318.1276 | 92.2 | 186.0 | 228.9 | 307.1214 | 239.7 | 94.3 | 595.3 |
| 319.1355 | 0.0 | 2.6 | 152.3 | 309.0938 | 168.5 | 96.6 | 311.8 |
| 320.1182 | 58.9 | 204.6 | 396.0 | 315.1040 | 248.9 | 12.3 | 15.3 |
| 326.1471 | 34.8 | 33.5 | 15.6 | 316.1120 | 315.9 | 33.8 | 0.0 |
| 328.1513 | 24.0 | 32.1 | 10.5 | 317.1198 | 1171.9 | 164.9 | 45.0 |
| 346.1169 | 11.6 | 105.4 | 228.1 | 318.1230 | 271.5 | 18.9 | 0.0 |
| 347.1026 | 0.0 | 0.0 | 372.1 | 325.1321 | 5.3 | 20.5 | 161.0 |

| | | | | | | | |
|---|---|---|---|---|---|---|---|
| 349.1359 | 3.0 | 62.4 | 154.0 | 327.1027 | 249.5 | 136.9 | 112.3 |
| 351.1065 | 157.7 | 268.0 | 641.0 | 334.0937 | 409.3 | 148.6 | 293.8 |
| 353.0783 | 38.0 | 0.0 | 2.6 | 337.1140 | 42.4 | 310.4 | 45.4 |
| 354.1141 | 8.5 | 41.7 | 118.8 | 340.1329 | 1.3 | 560.5 | 0.0 |
| 357.1147 | 76.8 | 99.1 | 25.9 | 346.0987 | 536.8 | 40.6 | 0.0 |
| 359.1186 | 146.0 | 139.3 | 60.8 | 351.1061 | 65.3 | 35.8 | 151.7 |
| 370.1179 | 176.3 | 396.9 | 972.0 | 353.1104 | 404.1 | 119.7 | 535.7 |
| 371.1210 | 1.2 | 35.9 | 164.8 | 358.1155 | 378.1 | 103.3 | 377.0 |
| 375.1120 | 5.0 | 27.8 | 15.0 | 370.1174 | 176.0 | 45.9 | 177.0 |
| 376.1262 | 229.5 | 271.3 | 156.7 | 379.1430 | 427.6 | 572.9 | 233.3 |
| 392.1146 | 54.9 | 92.0 | 25.7 | 381.0858 | 196.2 | 127.9 | 419.2 |
| 394.1261 | 28.7 | 204.6 | 169.4 | 382.1339 | 60.2 | 58.3 | 561.8 |
| 398.0703 | 103.7 | 23.3 | 0.0 | 400.1445 | 0.0 | 29.2 | 236.3 |
| 413.0494 | 115.1 | 41.9 | 0.0 | 411.1581 | 30.9 | 51.6 | 244.0 |
| 419.1135 | 0.0 | 0.0 | 161.5 | 415.1606 | 51.8 | 823.7 | 0.0 |
| 425.1082 | 192.7 | 223.6 | 110.4 | 416.1641 | 0.0 | 238.0 | 0.0 |
| 439.0835 | 212.3 | 157.6 | 80.1 | 452.1572 | 49.3 | 33.9 | 266.3 |
| 467.1542 | 102.3 | 170.8 | 204.6 | 457.1174 | 31.1 | 67.3 | 152.5 |
| 469.1542 | 109.7 | 235.9 | 81.1 | 458.1965 | 0.0 | 54.9 | 1429.1 |
| 470.1576 | 3.8 | 29.7 | 6.4 | 460.1359 | 136.7 | 105.9 | 284.4 |
| 475.1017 | 298.5 | 105.7 | 0.0 | 461.1484 | 59.8 | 322.4 | 57.1 |
| 481.1701 | 190.0 | 182.6 | 153.3 | 462.2051 | 0.0 | 46.6 | 1439.0 |
| 482.1782 | 380.8 | 442.6 | 417.1 | 465.1407 | 43.4 | 47.4 | 165.6 |
| 483.1810 | 103.4 | 145.4 | 132.4 | 465.1717 | 192.2 | 96.1 | 683.4 |
| 487.2038 | 304.3 | 365.3 | 219.5 | 468.1546 | 114.9 | 79.4 | 398.8 |
| 489.0803 | 274.7 | 129.3 | 0.0 | 475.1275 | 174.1 | 139.4 | 317.2 |
| 491.0963 | 178.0 | 131.8 | 0.0 | 479.1401 | 145.2 | 109.8 | 187.6 |
| 492.2129 | 26.1 | 258.5 | 494.9 | 479.1500 | 219.5 | 131.0 | 301.9 |
| 497.2012 | 0.0 | 36.7 | 287.5 | 482.1775 | 432.0 | 37.6 | 27.0 |
| 498.2093 | 85.7 | 148.5 | 51.3 | 483.1467 | 42.3 | 17.5 | 138.7 |
| 499.1149 | 118.3 | 58.5 | 12.9 | 483.1853 | 1010.5 | 129.8 | 90.6 |
| 507.0911 | 321.3 | 128.6 | 0.0 | 484.1553 | 135.5 | 87.9 | 389.6 |
| 510.1104 | 367.3 | 295.8 | 56.5 | 485.1671 | 42.5 | 342.6 | 57.7 |
| 517.1312 | 43.0 | 86.5 | 0.0 | 487.2031 | 457.9 | 49.6 | 60.4 |
| 539.1343 | 51.1 | 155.6 | 0.0 | 491.2090 | 800.0 | 35.8 | 0.0 |
| 542.1681 | 169.5 | 95.1 | 0.0 | 492.2129 | 260.9 | 0.0 | 0.0 |
| 551.1324 | 106.7 | 26.7 | 0.0 | 507.1701 | 155.8 | 417.9 | 205.3 |
| 561.2331 | 16.8 | 71.5 | 107.1 | 516.1494 | 129.9 | 52.0 | 45.4 |
| 571.1504 | 194.3 | 331.3 | 431.4 | 520.1770 | 0.0 | 171.8 | 23.5 |
| 573.1259 | 148.7 | 84.6 | 65.5 | 526.1390 | 111.6 | 108.3 | 254.2 |
| 574.1304 | 99.1 | 77.8 | 18.7 | 532.1941 | 20.8 | 1.0 | 334.5 |
| 574.1397 | 339.0 | 300.1 | 216.3 | 533.1956 | 204.3 | 79.9 | 1138.8 |
| 575.6534 | 241.3 | 234.7 | 148.0 | 534.1990 | 8.3 | 7.8 | 332.7 |
| 577.1674 | 60.5 | 261.0 | 53.6 | 539.1764 | 214.0 | 98.9 | 9.2 |
| 578.1612 | 346.7 | 223.1 | 77.8 | 539.1865 | 468.4 | 228.3 | 84.3 |

| | | | | | | | |
|---|---|---|---|---|---|---|---|
| 578.1705 | 0.8 | 58.8 | 0.0 | 541.1633 | 136.4 | 43.3 | 220.3 |
| 584.1312 | 129.6 | 89.4 | 0.0 | 541.1709 | 326.0 | 164.4 | 623.0 |
| 591.1464 | 361.4 | 275.2 | 23.6 | 541.2301 | 79.7 | 82.1 | 361.0 |
| 593.0674 | 179.9 | 102.3 | 0.0 | 542.1672 | 83.9 | 31.7 | 183.9 |
| 593.2557 | 174.3 | 82.7 | 0.0 | 542.1740 | 131.7 | 42.6 | 221.5 |
| 597.1740 | 224.3 | 169.3 | 31.7 | 542.1783 | 220.8 | 66.3 | 328.1 |
| 600.1735 | 144.8 | 123.7 | 8.6 | 546.1879 | 368.4 | 166.3 | 751.7 |
| 601.1856 | 7.1 | 49.6 | 0.0 | 546.1974 | 178.9 | 117.7 | 479.4 |
| 603.1658 | 0.0 | 0.0 | 0.0 | 552.1669 | 72.8 | 947.0 | 257.7 |
| 609.1361 | 60.3 | 0.0 | 34.9 | 553.1702 | 0.0 | 304.3 | 43.5 |
| 610.2592 | 30.3 | 0.0 | 0.0 | 559.1836 | 285.1 | 492.9 | 546.9 |
| 611.1730 | 308.1 | 271.7 | 100.5 | 561.1133 | 25.9 | 45.0 | 218.1 |
| 612.1755 | 43.3 | 62.2 | 9.5 | 561.2849 | 26.2 | 49.9 | 318.3 |
| 612.2630 | 39.6 | 0.0 | 0.0 | 565.2016 | 412.5 | 208.8 | 919.1 |
| 615.1658 | 3.2 | 40.8 | 0.0 | 565.2107 | 67.9 | 51.3 | 245.3 |
| 617.1362 | 368.2 | 698.8 | 425.7 | 568.1616 | 109.0 | 177.4 | 388.0 |
| 618.1399 | 85.5 | 243.8 | 101.6 | 575.2146 | 28.4 | 38.1 | 143.0 |
| 619.1337 | 60.4 | 197.7 | 87.3 | 578.2079 | 72.7 | 16.8 | 140.9 |
| 629.1724 | 116.9 | 18.5 | 35.3 | 583.2015 | 12.4 | 302.7 | 69.6 |
| 631.1860 | 59.6 | 65.6 | 0.0 | 583.2195 | 0.0 | 34.4 | 181.2 |
| 641.1560 | 50.6 | 74.6 | 0.0 | 588.2458 | 49.4 | 49.1 | 250.7 |
| 642.1850 | 36.2 | 0.0 | 13.8 | 595.2008 | 12.4 | 688.9 | 42.9 |
| 642.2704 | 0.0 | 64.6 | 403.5 | 596.2042 | 0.0 | 241.6 | 13.6 |
| 643.2746 | 0.0 | 7.2 | 154.9 | 599.2132 | 82.6 | 319.1 | 195.1 |
| 644.1560 | 256.2 | 94.7 | 61.7 | 600.2160 | 4.8 | 180.3 | 37.1 |
| 644.1637 | 92.5 | 68.9 | 22.9 | 609.1927 | 45.6 | 49.8 | 221.9 |
| 646.1780 | 240.7 | 55.5 | 0.0 | 609.2574 | 0.0 | 0.0 | 898.1 |
| 647.1649 | 89.6 | 67.7 | 13.6 | 612.1999 | 45.9 | 90.6 | 203.1 |
| 650.1726 | 199.5 | 52.9 | 41.9 | 613.2110 | 248.9 | 404.1 | 500.8 |
| 652.1861 | 163.3 | 179.2 | 0.0 | 613.2661 | 0.0 | 0.0 | 1045.8 |
| 667.1963 | 49.6 | 0.0 | 8.6 | 614.2143 | 59.3 | 177.7 | 157.7 |
| 669.1940 | 117.9 | 136.4 | 0.0 | 616.2017 | 252.5 | 175.7 | 616.6 |
| 671.1914 | 7.8 | 16.7 | 4.5 | 616.2768 | 21.5 | 27.1 | 130.7 |
| 685.2409 | 15.3 | 33.0 | 123.3 | 617.2052 | 39.7 | 78.8 | 195.7 |
| 697.1894 | 227.5 | 196.8 | 34.6 | 620.2222 | 108.3 | 676.3 | 231.0 |
| 702.1945 | 101.5 | 93.8 | 15.7 | 628.2042 | 118.1 | 61.9 | 321.9 |
| 703.1972 | 36.1 | 23.5 | 10.8 | 637.2300 | 138.8 | 772.7 | 245.2 |
| 705.2116 | 4.4 | 6.4 | 0.0 | 638.2333 | 23.8 | 342.5 | 60.9 |
| 718.1889 | 159.4 | 82.0 | 37.0 | 664.2216 | 403.0 | 372.4 | 575.2 |
| 718.1966 | 216.6 | 419.1 | 611.1 | 665.2248 | 164.5 | 160.2 | 229.8 |
| 723.1961 | 83.7 | 34.9 | 0.0 | 667.1963 | 107.6 | 190.5 | 197.1 |
| 726.0853 | 90.1 | 0.0 | 0.0 | 673.2470 | 0.0 | 1358.1 | 24.0 |
| 726.3392 | 71.4 | 0.0 | 0.0 | 674.2507 | 0.0 | 655.8 | 3.0 |
| 730.1335 | 10.7 | 76.3 | 263.0 | 683.1913 | 194.2 | 294.9 | 477.6 |
| 731.1276 | 17.3 | 68.2 | 194.1 | 683.2550 | 0.0 | 0.0 | 334.0 |

| | | | | | | | |
|---|---|---|---|---|---|---|---|
| 731.1409 | 13.8 | 77.5 | 245.7 | 684.1945 | 15.7 | 146.3 | 157.8 |
| 732.1357 | 74.3 | 196.1 | 435.5 | 684.2559 | 105.9 | 61.8 | 955.5 |
| 732.1491 | 0.0 | 19.3 | 128.1 | 685.2602 | 2.6 | 0.0 | 355.8 |
| 732.1863 | 181.2 | 132.7 | 4.1 | 686.2256 | 146.0 | 88.9 | 336.6 |
| 733.1433 | 56.6 | 197.3 | 435.5 | 686.2329 | 457.7 | 68.7 | 143.2 |
| 733.1579 | 24.3 | 134.6 | 936.6 | 689.2400 | 706.8 | 113.7 | 308.6 |
| 734.1515 | 15.1 | 81.4 | 258.7 | 691.2485 | 47.8 | 180.2 | 0.0 |
| 734.1609 | 0.0 | 0.2 | 300.2 | 692.2339 | 78.7 | 72.5 | 342.0 |
| 736.1636 | 0.0 | 48.1 | 582.0 | 693.2362 | 19.6 | 31.7 | 119.6 |
| 742.2061 | 55.0 | 52.6 | 0.5 | 696.2464 | 300.4 | 86.8 | 604.2 |
| 748.2160 | 199.5 | 0.6 | 0.0 | 697.2488 | 152.2 | 47.7 | 359.0 |
| 749.2197 | 47.4 | 12.1 | 0.0 | 697.2587 | 194.9 | 79.9 | 453.8 |
| 750.1564 | 51.5 | 170.9 | 414.2 | 717.2198 | 419.9 | 290.2 | 727.8 |
| 752.1584 | 110.8 | 397.9 | 709.8 | 718.2225 | 156.1 | 158.7 | 329.0 |
| 753.2770 | 0.0 | 0.0 | 187.1 | 723.2588 | 274.2 | 140.3 | 920.9 |
| 756.1865 | 212.1 | 64.8 | 31.2 | 724.2611 | 91.0 | 52.9 | 433.0 |
| 757.1908 | 47.0 | 11.0 | 6.1 | 727.2319 | 0.3 | 197.1 | 0.0 |
| 757.2020 | 103.8 | 46.9 | 12.7 | 738.2216 | 544.8 | 164.4 | 499.4 |
| 757.2987 | 0.0 | 30.8 | 392.8 | 739.2242 | 98.0 | 34.1 | 64.7 |
| 766.1414 | 1.0 | 0.0 | 136.9 | 741.2381 | 14.4 | 218.5 | 11.4 |
| 767.1500 | 0.0 | 81.2 | 160.7 | 744.2309 | 121.2 | 742.5 | 195.9 |
| 769.1659 | 0.0 | 30.9 | 173.2 | 745.2772 | 18.1 | 379.7 | 0.0 |
| 771.1884 | 281.9 | 154.6 | 0.0 | 746.2804 | 0.7 | 208.1 | 0.0 |
| 776.1939 | 36.2 | 55.2 | 0.0 | 759.2225 | 46.2 | 384.1 | 105.6 |
| 778.2267 | 73.5 | 0.0 | 17.6 | 760.2259 | 13.8 | 187.6 | 27.0 |
| 781.2087 | 153.6 | 76.0 | 5.0 | 761.2553 | 118.3 | 51.8 | 431.7 |
| 781.2351 | 47.8 | 0.0 | 14.6 | 762.2584 | 38.8 | 14.9 | 158.4 |
| 798.2096 | 137.9 | 151.8 | 105.4 | 765.3297 | 0.0 | 0.0 | 331.0 |
| 801.2158 | 79.4 | 93.8 | 67.8 | 767.2457 | 718.0 | 116.4 | 571.3 |
| 802.3219 | 0.0 | 0.0 | 167.7 | 767.2799 | 216.0 | 29.7 | 0.0 |
| 803.2467 | 250.3 | 217.6 | 12.7 | 768.2493 | 330.6 | 39.3 | 245.9 |
| 836.1752 | 0.0 | 37.3 | 134.6 | 781.2601 | 0.0 | 252.3 | 0.0 |
| 838.1775 | 14.6 | 121.2 | 269.2 | 793.2511 | 261.0 | 267.1 | 409.3 |
| 838.4042 | 38.7 | 0.0 | 7.9 | 794.2536 | 110.8 | 160.4 | 204.3 |
| 839.2336 | 13.7 | 80.4 | 101.7 | 798.2763 | 353.8 | 108.4 | 484.7 |
| 851.2827 | 49.1 | 223.2 | 300.6 | 799.2609 | 94.7 | 201.8 | 199.3 |
| 852.2867 | 5.4 | 100.0 | 175.4 | 801.2752 | 9.4 | 296.2 | 71.6 |
| 853.2373 | 0.0 | 32.1 | 52.9 | 813.2501 | 79.6 | 129.2 | 55.3 |
| 855.2592 | 9.7 | 0.1 | 0.6 | 818.2575 | 68.2 | 111.0 | 121.9 |
| 857.2917 | 219.9 | 381.7 | 341.8 | 820.2746 | 117.4 | 116.0 | 0.0 |
| 858.2946 | 108.2 | 234.7 | 176.4 | 823.2811 | 335.2 | 454.0 | 0.0 |
| 859.2197 | 125.4 | 219.4 | 164.3 | 834.3161 | 0.0 | 10.4 | 696.8 |
| 859.7209 | 143.6 | 190.1 | 179.8 | 836.3215 | 0.0 | 21.9 | 749.9 |
| 860.2226 | 58.8 | 91.2 | 77.1 | 839.2630 | 211.6 | 101.5 | 523.5 |
| 863.2418 | 0.0 | 7.0 | 222.3 | 840.2656 | 60.8 | 20.5 | 213.8 |

| | | | | | | | |
|---|---|---|---|---|---|---|---|
| 865.2019 | 67.6 | 73.0 | 0.0 | 844.3085 | 251.2 | 77.8 | 691.4 |
| 865.4311 | 48.0 | 0.0 | 8.7 | 847.3172 | 122.7 | 50.7 | 477.8 |
| 866.2153 | 55.3 | 83.3 | 50.6 | 855.3029 | 91.6 | 698.4 | 172.6 |
| 868.2423 | 150.5 | 122.7 | 119.0 | 856.3057 | 14.2 | 474.2 | 64.2 |
| 870.2539 | 355.7 | 397.9 | 553.8 | 871.2970 | 90.5 | 383.5 | 133.6 |
| 871.2278 | 300.3 | 211.4 | 68.7 | 872.3003 | 34.5 | 252.6 | 65.6 |
| 871.2555 | 51.6 | 48.4 | 106.1 | 873.2863 | 0.0 | 266.8 | 0.0 |
| 872.2293 | 119.2 | 77.4 | 6.7 | 877.3064 | 118.0 | 272.8 | 363.3 |
| 874.2566 | 60.3 | 33.5 | 16.2 | 878.3097 | 20.3 | 175.9 | 193.9 |
| 875.2720 | 261.8 | 131.8 | 43.9 | 888.2809 | 815.6 | 32.4 | 217.4 |
| 903.2034 | 181.8 | 0.0 | 47.1 | 889.2835 | 442.0 | 61.0 | 82.8 |
| 904.2075 | 87.9 | 0.0 | 22.9 | 894.2893 | 80.7 | 97.0 | 23.0 |
| 915.3888 | 14.8 | 0.0 | 0.0 | 895.2894 | 41.5 | 291.2 | 21.4 |
| 919.2426 | 0.0 | 37.9 | 341.2 | 897.3056 | 588.2 | 612.9 | 0.0 |
| 922.2505 | 32.8 | 212.5 | 918.5 | 898.3089 | 325.9 | 399.1 | 0.0 |
| 925.2373 | 33.3 | 30.2 | 0.0 | 910.3785 | 0.0 | 0.0 | 327.6 |
| 925.2505 | 97.8 | 85.9 | 40.8 | 913.2818 | 24.3 | 33.1 | 143.1 |
| 927.2037 | 0.0 | 39.6 | 89.1 | 916.3910 | 0.0 | 0.0 | 763.6 |
| 928.2576 | 147.5 | 132.4 | 58.5 | 917.2585 | 242.5 | 186.9 | 508.1 |
| 935.2244 | 287.9 | 165.0 | 13.5 | 918.2619 | 139.0 | 132.6 | 324.5 |
| 936.2286 | 165.5 | 59.6 | 0.0 | 925.2827 | 181.5 | 206.8 | 371.3 |
| 945.2550 | 53.5 | 172.8 | 192.1 | 926.2860 | 70.2 | 152.5 | 181.3 |
| 949.2578 | 118.3 | 63.9 | 52.9 | 941.2775 | 63.0 | 509.4 | 76.4 |
| 989.2104 | 145.4 | 145.5 | 35.0 | 942.2797 | 14.1 | 326.6 | 34.3 |
| 990.2127 | 62.9 | 76.5 | 6.4 | 970.3203 | 44.4 | 39.6 | 104.2 |
| 1009.2693 | 27.8 | 215.8 | 652.3 | 979.3098 | 58.9 | 350.4 | 9.8 |
| 1010.2720 | 67.6 | 191.4 | 226.2 | 980.3136 | 24.8 | 267.9 | 0.0 |
| 1015.2799 | 323.3 | 556.4 | 580.8 | 985.3183 | 0.0 | 199.3 | 0.1 |
| 1019.2789 | 13.9 | 219.5 | 143.1 | 986.3792 | 0.0 | 0.0 | 547.0 |
| 1020.2835 | 0.0 | 126.3 | 79.7 | 991.4116 | 278.2 | 2.9 | 0.0 |
| 1031.3454 | 55.6 | 30.0 | 0.0 | 992.4089 | 614.0 | 16.6 | 0.0 |
| 1036.2329 | 0.0 | 20.2 | 114.5 | 993.2898 | 95.3 | 171.7 | 177.5 |
| 1049.2464 | 2.8 | 7.5 | 0.0 | 993.4139 | 546.6 | 12.3 | 0.0 |
| 1061.2393 | 256.2 | 136.4 | 8.1 | 994.2920 | 52.4 | 122.7 | 135.7 |
| 1061.3531 | 0.0 | 102.3 | 558.2 | 994.3699 | 42.1 | 23.4 | 520.2 |
| 1066.2428 | 126.5 | 209.2 | 0.0 | 998.3053 | 167.7 | 40.3 | 250.2 |
| 1067.2543 | 16.8 | 78.1 | 5.9 | 998.3159 | 441.1 | 181.6 | 709.8 |
| 1068.2603 | 0.2 | 37.8 | 0.0 | 999.3108 | 81.7 | 10.7 | 123.4 |
| 1074.2666 | 257.7 | 207.5 | 0.0 | 999.3809 | 107.2 | 22.5 | 756.2 |
| 1077.3137 | 299.8 | 310.7 | 187.7 | 1003.3205 | 99.2 | 208.8 | 205.5 |
| 1078.3149 | 184.4 | 165.2 | 110.3 | 1003.3632 | 8.1 | 280.9 | 0.0 |
| 1088.2587 | 34.3 | 86.2 | 0.0 | 1004.3314 | 72.7 | 162.7 | 0.0 |
| 1091.2663 | 117.2 | 186.2 | 23.0 | 1004.3657 | 0.0 | 224.1 | 0.0 |
| 1094.3009 | 130.4 | 68.9 | 0.0 | 1017.3092 | 16.8 | 689.4 | 65.0 |
| 1127.2921 | 0.0 | 275.6 | 58.5 | 1018.3108 | 14.1 | 516.4 | 45.8 |

| | | | | | | | |
|---|---|---|---|---|---|---|---|
| 1128.2950 | 4.2 | 201.9 | 34.7 | 1019.3249 | 7.2 | 169.1 | 39.8 |
| 1139.2860 | 98.7 | 261.3 | 612.6 | 1020.3273 | 5.9 | 159.3 | 13.9 |
| 1140.2713 | 68.8 | 87.3 | 12.9 | 1027.3814 | 433.6 | 0.0 | 0.0 |
| 1148.3042 | 440.2 | 403.6 | 268.8 | 1028.3847 | 316.2 | 0.0 | 0.0 |
| 1149.2960 | 229.6 | 148.2 | 0.0 | 1055.3417 | 0.0 | 550.4 | 46.7 |
| 1150.2995 | 177.7 | 108.8 | 88.7 | 1056.3447 | 0.0 | 465.5 | 39.9 |
| 1151.3069 | 22.1 | 50.4 | 36.4 | 1059.3923 | 172.7 | 26.1 | 232.7 |
| 1165.2895 | 38.0 | 158.6 | 0.0 | 1060.3941 | 114.3 | 17.1 | 189.4 |
| 1166.2927 | 24.0 | 89.0 | 0.0 | 1063.4429 | 0.0 | 0.0 | 935.0 |
| 1185.2718 | 110.9 | 329.6 | 192.9 | 1065.4454 | 0.0 | 0.0 | 1011.1 |
| 1186.2762 | 65.9 | 208.7 | 127.0 | 1066.4487 | 0.0 | 0.0 | 287.1 |
| 1194.2988 | 57.6 | 0.3 | 14.5 | 1071.3360 | 518.3 | 439.3 | 556.0 |
| 1210.2930 | 373.6 | 470.9 | 374.6 | 1072.3407 | 474.3 | 362.7 | 453.4 |
| 1211.2953 | 304.6 | 392.8 | 289.5 | 1073.3514 | 1208.3 | 157.9 | 52.5 |
| 1212.2932 | 168.0 | 99.1 | 83.4 | 1074.3541 | 1040.3 | 159.1 | 10.3 |
| 1213.2952 | 101.0 | 0.2 | 28.1 | 1075.3583 | 360.3 | 53.3 | 0.0 |
| 1216.3028 | 320.5 | 277.5 | 134.9 | 1076.3424 | 0.0 | 125.6 | 65.4 |
| 1217.3041 | 263.5 | 255.5 | 129.2 | 1085.3693 | 68.8 | 13.0 | 115.2 |
| 1218.3065 | 73.8 | 63.2 | 12.3 | 1093.3382 | 0.0 | 274.1 | 12.4 |
| 1219.3188 | 184.6 | 337.5 | 47.1 | 1094.3423 | 0.0 | 195.5 | 2.6 |
| 1220.3209 | 135.1 | 273.6 | 45.0 | 1094.4664 | 1964.9 | 0.0 | 0.0 |
| 1221.3271 | 38.7 | 42.5 | 0.0 | 1095.3551 | 74.9 | 312.4 | 17.9 |
| 1287.3314 | 0.0 | 75.2 | 232.1 | 1096.3571 | 44.7 | 275.4 | 2.9 |
| 1288.3346 | 0.0 | 47.5 | 199.5 | 1100.4721 | 638.5 | 0.0 | 0.0 |
| 1298.3623 | 143.0 | 138.0 | 117.1 | 1131.3729 | 0.0 | 209.8 | 0.0 |
| 1301.2944 | 0.0 | 57.0 | 357.5 | 1145.4304 | 0.0 | 0.0 | 352.6 |
| 1302.2950 | 0.0 | 10.8 | 268.0 | 1146.3663 | 585.3 | 85.5 | 412.3 |
| 1303.2968 | 19.0 | 215.4 | 738.4 | 1147.3686 | 508.7 | 147.4 | 340.9 |
| 1304.2997 | 9.8 | 97.0 | 528.2 | 1148.3722 | 141.2 | 87.1 | 82.0 |
| 1305.3038 | 0.0 | 0.0 | 135.1 | 1150.4411 | 30.0 | 0.6 | 526.5 |
| 1326.3390 | 109.3 | 56.3 | 38.0 | 1153.3759 | 12.3 | 335.3 | 58.8 |
| 1346.3616 | 29.5 | 0.0 | 14.7 | 1154.3795 | 0.0 | 230.1 | 10.5 |
| 1349.3713 | 76.8 | 0.0 | 33.0 | 1156.3959 | 399.1 | 1028.7 | 260.8 |
| 1367.3473 | 223.1 | 366.7 | 295.4 | 1157.3981 | 108.4 | 398.3 | 53.2 |
| 1368.3502 | 176.9 | 239.9 | 266.6 | 1215.5050 | 0.0 | 0.0 | 297.2 |
| 1369.3508 | 46.5 | 75.7 | 52.6 | 1229.4217 | 1020.6 | 9.3 | 724.9 |
| 1437.3873 | 11.3 | 16.6 | 106.6 | 1233.4293 | 837.1 | 60.2 | 577.0 |
| 1438.3913 | 5.2 | 20.9 | 103.9 | 1234.4314 | 131.6 | 7.1 | 85.0 |
| 1450.4254 | 127.1 | 46.0 | 20.0 | 1296.4905 | 0.0 | 0.0 | 269.2 |
| 1481.5206 | 85.4 | 0.0 | 0.0 | 1301.5026 | 0.0 | 0.0 | 442.0 |
| 1482.5213 | 40.8 | 0.0 | 0.0 | 1447.5526 | 0.0 | 0.0 | 290.5 |

# Supplementary References

1. Ninomiya, S. *et al.* Precise and fast secondary ion mass spectrometry depth profiling of polymer materials with large Ar cluster ion beams. *Rapid Commun. Mass Spectrom.* **23**, (2009).
2. Giebink, N. C. *et al.* Intrinsic luminance loss in phosphorescent small-molecule organic light emitting devices due to bimolecular annihilation reactions. *J. Appl. Phys.* **103**, (2008).
3. Trindade, G. F., Abel, M. L. & Watts, J. F. simsMVA: A tool for multivariate analysis of ToF-SIMS datasets. *Chemom. Intell. Lab. Syst.* **182**, 180–187 (2018).
4. Shim, S., Choi, E., Kim, H. & Yun, J. Y. Degradation Behaviors of NPB Molecules upon Prolonged Exposure to Various Thermal Stresses under High Vacuum below 10-4Pa. *ACS Omega* **5**, (2020).
