# Supplementary material for: Direct identification of interfacial degradation in blue OLEDs using nanoscale chemical depth profiling
Source: Nat Commun. 2023 Dec 6;14:8066. doi: 10.1038/s41467-023-43840-9 (PMC10698160; doi:10.1038/s41467-023-43840-9)
Supplement: Supplementary file 3 — Related Manuscript File [file 41467_2023_43840_MOESM3_ESM.pptx]

## Slide 1
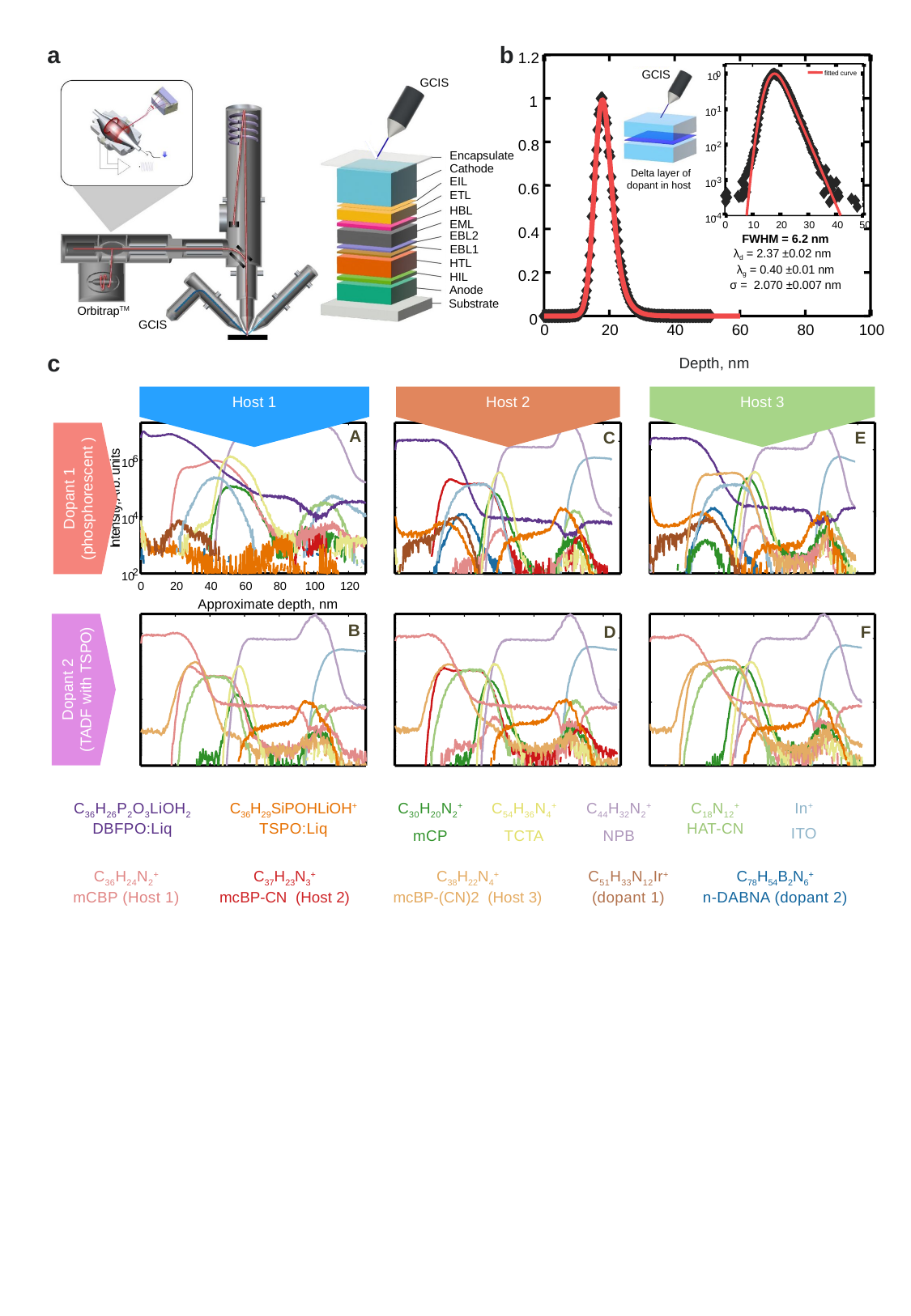

a
b
1.2
1
0.8
0.6
0.4
0.2
0
0
20
40
60
80
100
0
10
-1
10
-2
10
-3
10
-4
10
0
10
20
30
40
50
fitted curve
GCIS
GCIS
Delta layer of dopant in host
OrbitrapTM
GCIS
Encapsulate
Cathode
EIL
ETL
HBL
EML
EBL2
FWHM = 6.2 nm
λd = 2.37 ±0.02 nm
λg = 0.40 ±0.01 nm
σ = 2.070 ±0.007 nm
EBL1
HTL
HIL
Anode
Substrate
c
Depth, nm
Host 1
Host 2
Host 3
Dopant 1
(phosphorescent )
Dopant 2
(TADF with TSPO)
A
C
E
s
t
i
6
n
10
u
.
b
r
A
,
y
t
i
4
s
10
n
e
t
n
I
2
10
0
20
40
60
80
100
120
Approximate depth, nm
B
D
F
C36H26P2O3LiOH2 DBFPO:Liq
C36H29SiPOHLiOH+
TSPO:Liq
C30H20N2+
mCP
C54H36N4+
TCTA
C44H32N2+
NPB
C18N12+
HAT-CN
In+
ITO
C36H24N2+
mCBP (Host 1)
C37H23N3+
mcBP-CN (Host 2)
C38H22N4+
mcBP-(CN)2 (Host 3)
C51H33N12Ir+
(dopant 1)
C78H54B2N6+
n-DABNA (dopant 2)

## Slide 2
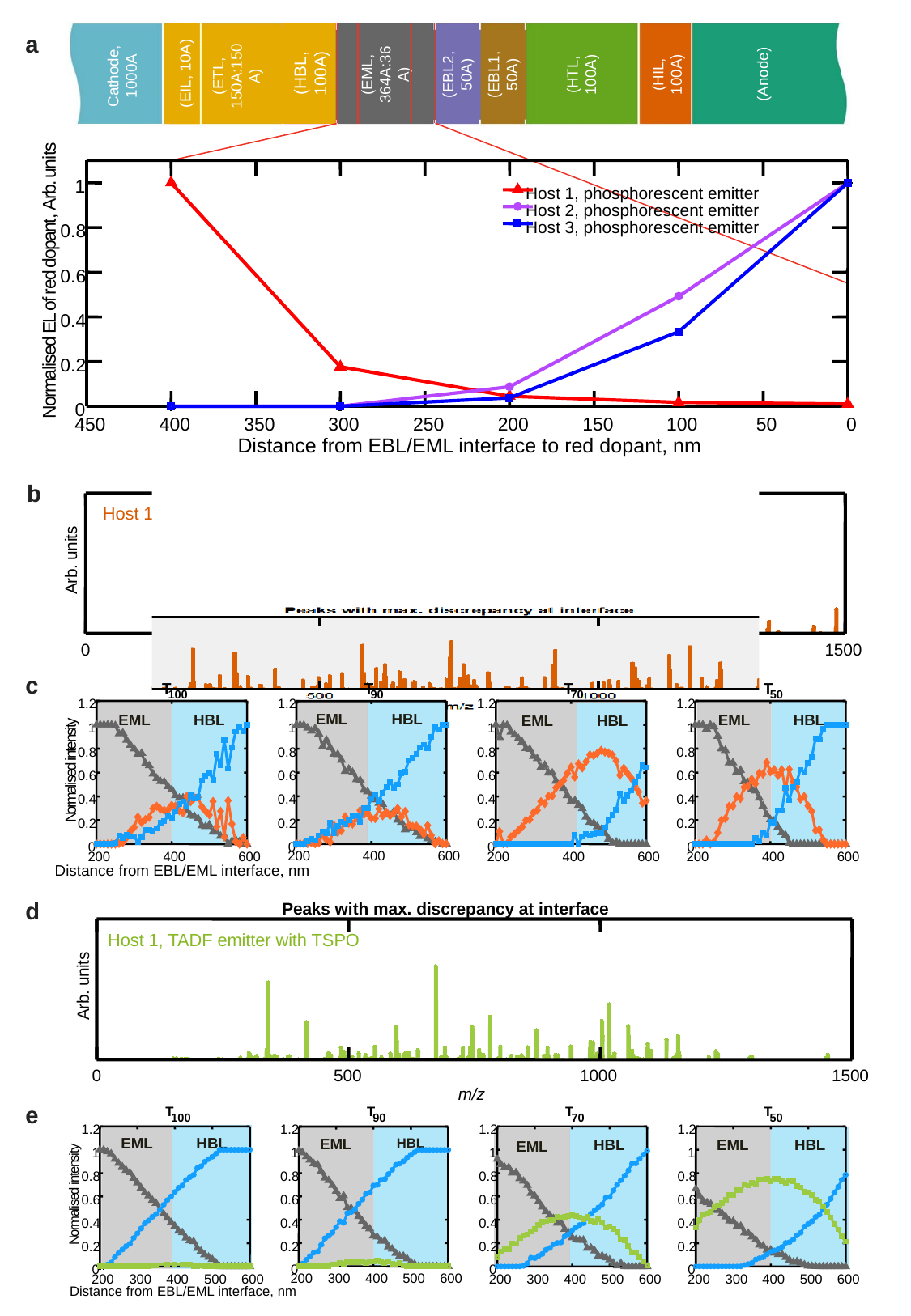

(EML, 364A:36A)
(ETL, 150A:150A)
(HBL, 100A)
(HIL, 100A)
(EBL2, 50A)
(EBL1, 50A)
(HTL, 100A)
Cathode, 1000A
(EIL, 10A)
(Anode)
a
s
t
i
n
u
.
1
b
Host 1, phosphorescent emitter
r
A
Host 2, phosphorescent emitter
,
t
Host 3, phosphorescent emitter
n
0.8
a
p
o
d
0.6
d
e
r
f
o
L
0.4
E
d
e
s
0.2
i
l
a
m
r
o
0
N
450
400
350
300
250
200
150
100
50
0
Distance from EBL/EML interface to red dopant, nm
Peaks with max. discrepancy at interface
s
t
i
n
u
.
b
r
A
0
500
1000
1500
m/z
b
Host 1, Phosphorescent emitter with DBFPO
c
T
100
1.2
y
t
1
i
s
n
e
t
0.8
n
i
d
e
0.6
s
i
l
a
m
0.4
r
o
N
0.2
0
200
400
600
200
400
600
Distance from EBL/EML interface, nm
T
T
90
70
1.2
1.2
1
1
0.8
0.8
0.6
0.6
0.4
0.4
0.2
0.2
0
0
200
400
600
T
50
1.2
1
0.8
0.6
0.4
0.2
0
200
400
600
EML
HBL
EML
HBL
EML
HBL
EML
HBL
d
Peaks with max. discrepancy at interface
s
t
i
n
u
.
b
r
A
0
500
1000
1500
m/z
Host 1, TADF emitter with TSPO
e
T
T
T
T
90
100
70
50
1.2
1.2
1.2
1.2
y
t
1
1
1
1
i
s
n
e
t
0.8
0.8
0.8
0.8
n
i
d
e
0.6
0.6
0.6
0.6
s
i
l
a
m
0.4
0.4
0.4
0.4
r
o
N
0.2
0.2
0.2
0.2
0
0
0
0
200
300
400
500
600
200
300
400
500
600
400
200
300
500
600
200
300
400
500
600
Distance from EBL/EML interface, nm
HBL
EML
HBL
EML
HBL
EML
EML
HBL

## Slide 3
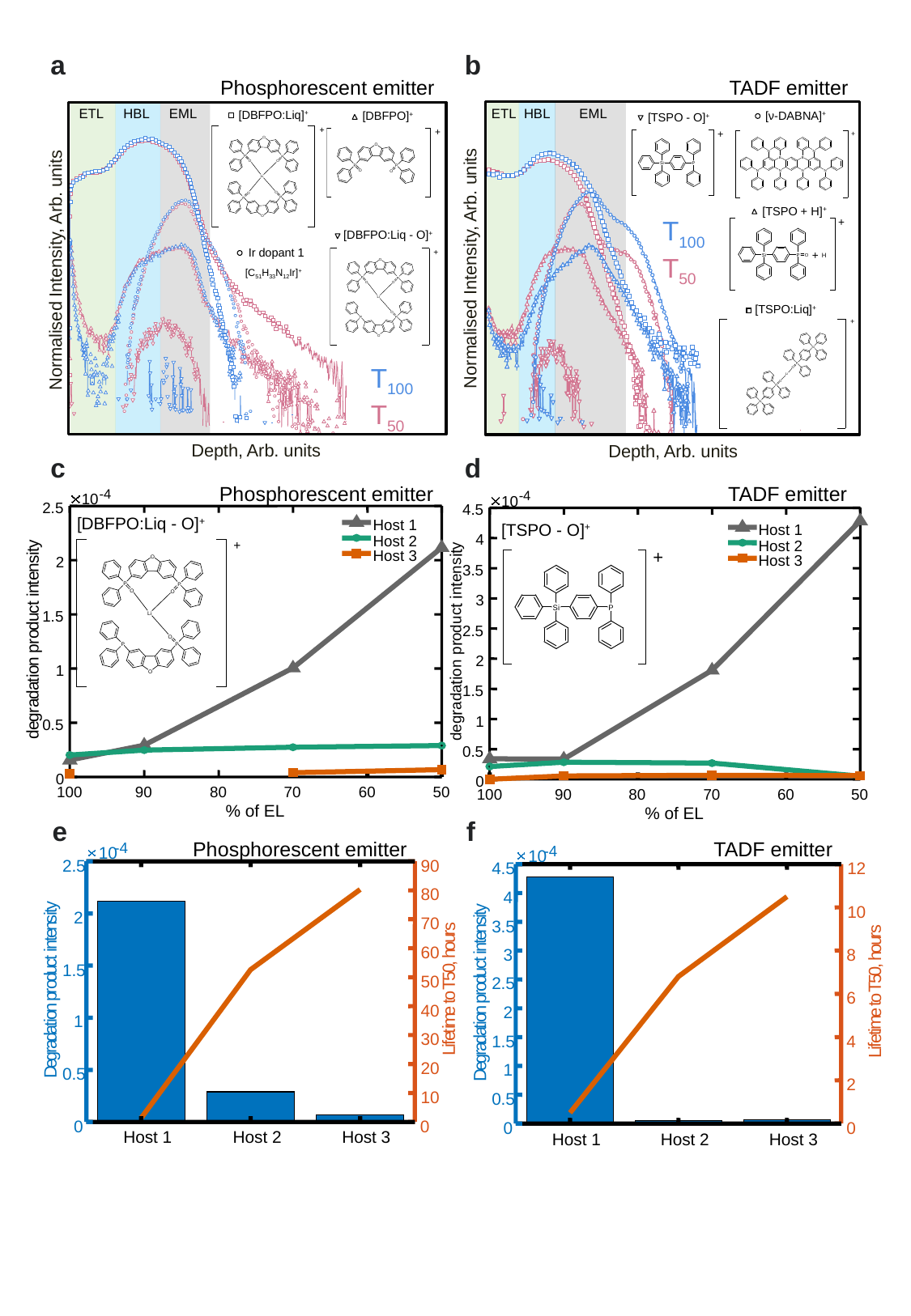

a
b
TADF emitter
Phosphorescent emitter
ETL
HBL
EML
ETL
HBL
EML
[DBFPO:Liq]+
[ν-DABNA]+
[DBFPO]+
[TSPO - O]+
[TSPO + H]+
T100
T50
[DBFPO:Liq - O]+
Ir dopant 1
[C51H33N12Ir]+
Normalised Intensity, Arb. units
Normalised Intensity, Arb. units
[TSPO:Liq]+
T100
T50
Depth, Arb. units
Depth, Arb. units
c
d
Phosphorescent emitter
TADF emitter
-4
-4
10
10
2.5
4.5
[DBFPO:Liq - O]+
[TSPO - O]+
Host 1
Host 1
4
Host 2
y
Host 2
t
y
t
i
s
n
e
t
n
i
t
c
u
d
o
r
p
n
o
i
t
a
d
a
r
g
e
d
i
Host 3
s
Host 3
2
n
3.5
e
t
n
i
3
t
c
1.5
u
d
2.5
o
r
p
2
n
o
1
i
t
a
1.5
d
a
r
g
1
0.5
e
d
0.5
0
0
100
90
80
70
60
50
100
90
80
70
60
50
% of EL
% of EL
e
f
Phosphorescent emitter
TADF emitter
-4
-4
10
4.5
12
4
y
10
t
i
s
3.5
n
s
r
e
u
t
n
o
i
3
8
h
t
c
,
0
u
5
d
2.5
o
T
r
6
o
p
t
n
2
e
o
i
m
t
i
a
t
e
d
1.5
4
f
a
i
r
L
g
e
1
D
2
0.5
0
0
Host 1
Host 2
Host 3
10
2.5
90
80
y
t
i
s
2
70
n
s
r
e
u
t
n
o
i
h
60
t
c
,
0
u
1.5
5
d
50
T
o
r
o
p
t
n
e
40
o
m
i
1
t
i
a
t
e
d
30
f
a
i
L
r
g
e
20
D
0.5
10
0
0
Host 1
Host 2
Host 3
